# Reduced prevalence of placental malaria in primiparae with blood group O

**DOI:** 10.1186/1475-2875-13-289

**Published:** 2014-07-28

**Authors:** George Bedu-Addo, Prabhanjan P Gai, Stefanie Meese, Teunis A Eggelte, Kumarasamy Thangaraj, Frank P Mockenhaupt

**Affiliations:** 1 Department of Medicine, Komfo Anoyke Teaching Hospital, School of Medical Sciences, Kwame Nkrumah University of Science and Technology, Kumasi, Ghana; 2 Institute of Tropical Medicine and International Health, Charité – University Medicine Berlin, Berlin, Germany; 3 Division of Infectious Diseases, Tropical Medicine and AIDS, Academic Medical Centre, Amsterdam, The Netherlands; 4 Centre for Cellular and Molecular Biology, Hyderabad, India

## Abstract

**Background:**

Blood group O protects African children against severe malaria and has reached high prevalence in malarious regions. However, its role in malaria in pregnancy is ambiguous. In 839 delivering Ghanaian women, associations of ABO blood groups with *Plasmodium falciparum* infection were examined.

**Methods:**

*Plasmodium falciparum* infection was diagnosed in placental blood samples by microscopy and PCR assays. Present or past infection was defined as the detection of parasitaemia or haemozoin by microscopy, or a positive PCR result. Blood groups were inferred from genotyping rs8176719 (indicating the O allele) and rs8176746/rs8176747 (distinguishing the B allele from the A allele).

**Results:**

The majority of women had blood group O (55.4%); present or past *P. falciparum* infection was seen in 62.3% of all women. Among multiparae, the blood groups had no influence on *P. falciparum* infection. In contrast, primiparae with blood group O had significantly less present or past infection than women with non-O blood groups (61.5 *vs* 76.2%, *P* = 0.007). In multivariate analysis, the odds of present or past placental *P. falciparum* infection were reduced by 45% in blood group O primiparae (aOR, 0.55 [95% CI, 0.33–0.94]).

**Conclusions:**

The present study shows a clear protective effect of blood group O against malaria in primiparae. This accords with findings in severe malaria and *in vitro* results. The data underline the relevance of host genetic protection among primiparae, i.e. the high-risk group for malaria in pregnancy, and contribute to the understanding of high O allele frequencies in Africa.

## Background

*Plasmodium falciparum* malaria has long been suggested to influence the global distribution of the ABO blood groups [1], similar to the selection of the malaria-protective sickle cell gene in endemic regions [2]. In fact, blood group O is particularly common in malarious regions, e.g. sub-Saharan Africa [3,4], and confers protection against potentially fatal severe malaria across African populations [5-8]. In addition, various *in vitro* observations and functional hypotheses support a protective role of blood group O (reviewed by [3,4,9]).

In contrast, findings are notably ambiguous with respect to the impact of the ABO system on malaria in pregnancy. Pregnant women – particularly primiparae – are a high risk group for *P. falciparum* infection and malaria. In areas of high transmission, malaria in pregnancy is frequently asymptomatic but consequences involve anaemia, abortion, stillbirth, low birth weight (LBW), preterm delivery (PTD), and, annually, up to 200,000 infant deaths [10]. In pregnant women, specific expression variants of the parasite’s *P. falciparum* erythrocyte membrane protein-1 (PfEMP1) mediate adhesion to the placental syncytiotrophoblast (the epithelial lining of the intervillous space) and thereby placental sequestration of infected erythrocytes, deposition of haemozoin (malaria pigment), and, commonly, the local accumulation of inflammatory cells [11]. In highly endemic regions, specific immunity against these pregnancy-associated parasites is particularly low in primigravidae, and acquired only with successive pregnancies, which goes along with declining infection prevalence and clinical manifestation [10-12]. In The Gambia and Malawi, blood group O was associated with increased odds of placental malaria in primiparae but with a reduced risk in multiparae [13,14]. In Sudan, blood group O and past placental infection were associated in both primi- and multiparae [15]. In contrast, a Gabonese study reported a trend towards less placental malaria in blood group O women [16], and in a recent prospective study from Thailand, no association at all between ABO blood groups and malaria during pregnancy was observed [17]. Against this background of conflicting results, the present study aimed at examining the influence of blood group O on *P. falciparum* infection among pregnant women in hyper- to holo-endemic Ghana using PCR for blood group genotyping.

## Methods

Eight-hundred and thirty-nine women with live singleton delivery were recruited from January 2000 through January 2001 at the Presbyterian Mission Hospital in Agogo, Ghana. The study protocol was approved by the Committee on Human Research Publications and Ethics, School of Medical Sciences, University of Science and Technology, Kumasi, and informed written consent was obtained from all women. Agogo is a community of some 30,000 inhabitants located in the forested hills of Asante Akim North District. Subsistence farming, trade and mining are the main income sources in that region, and malaria is hyper- to holo-endemic [18]. Study procedures and the characteristics of the largely asymptomatic participants have been described in detail elsewhere [12]. In brief, women were clinically examined, socio-economic data documented, and intervillous and venous blood samples were collected into EDTA. Malaria parasites in post-delivery placental and venous samples were counted microscopically on Giemsa-stained thick blood films *per* 100 high-power fields and 500 white blood cells, respectively. Placental parasite densities were expressed as parasites/100 fields and peripheral ones as parasites/μL assuming a mean white blood cell count of 8,000/μL. Leukocyte-associated haemozoin in placental samples was recorded. Following DNA extraction (QIAmp, Qiagen, Hilden, Germany), nested *P. falciparum*-specific PCR assays were performed [19]. Present or past placental *P. falciparum* infection was defined as the presence of placental parasitaemia or haemozoin by microscopy, or a positive *P. falciparum* PCR result on placental samples. Plasma concentrations of pyrimethamine, at that time recommended for malaria chemoprophylaxis in pregnancy, and of chloroquine were measured by ELISA assays [20] with limits of detection of 10 ng/mL and 5 ng/ml, respectively. Haemoglobin (Hb) was measured by a HemoCue photometer (Ångelholm, Sweden). Anaemia was defined as Hb <11 g/dL. Birth weight and gestational age were assessed within 24 hours after delivery. LBW was defined as a birth weight <2500 g. PTD was defined as a gestational age <37 weeks applying the Finnström score which correctly estimates gestational age (±3 weeks) in >95% of infants [21].

Three loci on the *ABO* gene, namely rs8176719, rs8176746, and rs8176747 were typed by melting curve analysis employing the LightCycler 480 device (Roche Diagnostics, Mannheim, Germany) and using commercially available primers and probes (TIB Molbiol, Berlin, Germany). ABO blood groups were inferred from rs8176719 (indicating the *O* allele) and from rs8176746/rs8176747 (distinguishing the *B* allele from the *A* allele) [22]. In case of ambiguous typing results (*n* = 38), PCR-generated fragments containing the polymorphisms of interest were subjected to sequencing (Eurofins, Berlin, Germany).

Continuous variables were compared between groups by *t*-test, analysis of variance, Mann–Whitney *U*-test, and Kruskal-Wallis test as applicable. Associations of blood groups with *P. falciparum* infection, anaemia, LBW, and PTD were identified by *χ*^2^-test, and odds ratios (ORs) and 95% confidence intervals (95% CIs) calculated. Adjusted ORs (aORs) were derived from logistic regression models with stepwise backward removal of factors not associated in multivariate analysis (*P* > 0.05). For placental malaria, previously identified associated factors were *a priori* included, i.e. delivery in rainy season, age and plasma pyrimethamine concentrations [12]. A *P*-value of <0.05 was considered statistically significant.

## Results

ABO genotyping was successful in 827 of 839 (98.6%) women with a live singleton delivery. The majority of women (55.4%) had blood group O. Blood groups B, A, and AB were present in 22.6, 18.0, and 4.0%, respectively. Blood groups were in Hardy-Weinberg equilibrium; genotypes are detailed in Table 1. Plasma concentrations of pyrimethamine, representing chemoprophylaxis, were observed in 36.0% (163/453) of women with blood group O, and in 33.3% (119/357) of women with non-O blood groups (*P* = 0.43; chloroquine plasma concentrations, 24.3% (110/453) *vs.* 20.2% (72/357), *P* = 0.16).

**Table 1:** ABO genotypes in 827 delivering Ghanaian women

**Phenotype**	**Genotype**	**No (%)**
O	O O*	458 (55.4)
B	B O	169 (20.4)
	B B	18 (2.2)
A	A O	136 (16.4)
	A A	13 (1.6)
AB	A B	33 (4.0)

*, includes genotypes OO^24^ (*n* = 30), O^24^O^24^ (*n* = 1), and O^2^O^2^ (*n* = 1).

Evidence of present or past *P. falciparum* infection in placental samples (parasitaemia or haemozoin by microscopy, or a positive *P. falciparum* PCR result) was seen in 62.3% (515/827) of all women. This figure was significantly lower in women with blood group O (59.0%, 270/458) than in women with non-O blood groups (66.4%, 245/369; *P* = 0.03). Notably, this difference was pronounced among primiparae (61.5% *vs.* 76.2%, *P* = 0.007) but insignificant among multiparae (Table 2). Further analysis therefore focused on the 300 primiparous women. Among these, the prevalence of infection (regardless of diagnostic tool) appeared to be lower in women with blood group O than in any of the non-O blood groups. Based on parasite detection by microscopy, this difference was only small. Likewise, neither placental nor peripheral blood parasite densities correlated with the blood groups (e.g., placental parasite densities in blood group O *versus* non-O primiparae: 127 (95% CI, 74–216) *vs.* 102 (51–204) parasites/100 high-power fields, *P* = 0.63). In contrast, the reduced infection prevalence among blood group O primiparae was obvious taking into account PCR results, and even more so when considering placental haemozoin (Table 3). In multivariate analysis, the odds of present or past placental *P. falciparum* infection were reduced by 45% in primiparae with blood group O (Table 3), adjusting for known predictors of placental infection in this group [12], i.e. years of age (aOR, 0.92 [95% CI, 0.86–0.99]), delivery in the rainy season (aOR, 1.76 [95% CI, 1.06–2.92]), and presence of pyrimethamine in plasma (indicating compliance with chemoprophylaxis; aOR, 0.60 [95% CI, 0.35–1.00]). Breakdown by placental infection status [23] revealed that blood group O tended to protect particularly against late infections (both parasites and pigment present), while - combining microscopy and PCR results - the impact on microscopic (i.e. visible parasites) and submicroscopic infections (i.e. detectable by PCR only) was similar (Table 3).

**Table 2:** **Prevalence of *Plasmodium falciparum *** **infection according to blood group and parity**

	**Blood group, primiparae**					**Blood group, multiparae**				
	**O**	**Non-O**				**O**	**Non-O**			
		**All**	**B**	**A**	**AB**		**All**	**B**	**A**	**AB**
No.	174	126	72	46	8	283	237	111	102	24
*P. falciparum* infection, placental blood (%)										
Microscopy positive	42.5	50.0	52.8	43.5	62.5	30.0	27.0	31.5	21.6	29.2
Haemozoin positive	36.2	50.8*	52.8*	50.0	37.5	24.0	24.9	28.8	17.6	37.5
PCR positive	60.3	70.6	65.3	76.1*	87.5	55.5	58.2	60.4	54.9	62.5
Any positive finding^†^	61.5	76.2*	69.4	84.8*	87.5	57.6	61.6	64.0	57.8	66.7
*P. falciparum* infection, peripheral blood (%)										
Microscopy positive	24.1	29.4	33.3	26.1	12.5	17.0	11.4	14.4	8.8*	8.3
PCR positive	54.6	65.1	62.5	69.6	62.5	50.5	49.4	52.3	45.1	54.2

*Comparison to blood group O, *P* < 0.05; ^†^, parasitaemia or haemozoin by microscopy, or positive PCR. Parity data were missing for seven women.

**Table 3:** **Placental *Plasmodium falciparum *** **infection in primiparae separated by blood group O (*n*** **= 174) vs non-O blood groups (*n*** **= 126)**

**Placental infection status**	**Blood group**		**OR (95% CI)**	***P***	aOR* (95% CI)	***P***
	**O**	**Non-O**				
Microscopy positive (%)	42.5	50.0	0.74 (0.45-1.20)	0.20	0.80 (0.49-1.28)	0.35
Haemozoin positive (%)	36.2	50.8	0.55 (0.34-0.90)	0.01	0.61 (0.37-0.98)	0.04
PCR positive (%)	60.3	70.6	0.63 (0.38-1.06)	0.07	0.68 (0.41-1.13)	0.14
Any positive finding (%)^†^	61.5	76.2	0.50 (0.29-0.86)	0.007	0.55 (0.33-0.94)	0.03
Microscopic infection status (%)						
Pigment only	5.2	8.7	0.47 (0.16-1.32)	0.11	0.62 (0.23-1.67)	0.35
Pigment *plus* parasites	31.0	42.1	0.58 (0.34-1.0)	0.04	0.64 (0.38-1.09)	0.10
Parasites only	11.5	7.9	1.14 (0.46-2.85)	0.75	1.33 (0.55-3.20)	0.52
PCR-based infection status (%)						
Submicroscopic	17.8	22.2	0.56 (0.28-1.14)	0.08	0.59 (0.30-1.14)	0.12
Microscopic	42.5	50.0	0.60 (0.34-1.04)	0.05	0.64 (0.37-1.11)	0.12

Odds ratios are calculated against the respective non-infected reference group.

*Adjusted for age, rainy season, and presence of pyrimethamine in plasma (indicating intake of chemoprophylaxis).

^†^Parasitaemia or haemozoin by microscopy, or positive PCR.

Small numbers prevented a meaningful attribution of infection risks to individual genotypes. Nevertheless, present or past placental infection was observed in 88.9% (16/18) of primiparae with homozygous non-O genotypes, in 74.1% (80/108) of O-heterozygous women, and in 61.5% (107/174) of first-time delivering women with blood group O (c^2^ trend = 8.7, *P* = 0.003). Correspondingly, the *O* allele was significantly less frequent in primiparae with present or past placental infection than in those without (0.72 *vs* 0.84, *P* = 0.003).

In multiparae, none of the above malaria-protective features of blood group O were discernible; blood group A even tended to come along with lower infection prevalence (Table 2). Particularly, already in secundiparae, present or past placental infection occurred at similar prevalence in women with blood group O and in the non-O group (66.0% (70/106) *vs* 69.2% (54/78), *P* = 0.65).

Blood group O was furthermore associated with reduced odds of maternal anaemia among all women, and among primiparae in particular (Table 4). This was due to the difference in women without placental malaria, i.e. only 10.5% (7/67) of non-malarious blood group O primiparae in this group were anaemic as compared to 33.3% (10/30) of their non-O peers (*P* = 0.006) whereas the difference was small and not significant among primiparae with evidence of present or past infection (44.9%, 48/107 *vs* 52.1%, 50/96, *P* = 0.30). Likewise, the proportions of malaria-associated LBW or preterm delivery did not differ significantly between O and non-O blood groups, neither in primiparae nor in multiparae.

**Table 4:** Anaemia, low birth weight, and preterm delivery separated by blood group and parity

**Parameter**	**All**		**Primiparae**		**Multiparae**	
	**Blood group O**	**Non-O blood groups**	**O**	**Non-O blood groups**	**O**	**Non-O blood groups**
Anaemia (%, n/n)	31.7 (145/458)	39.6 (146/369)*	31.6 (55/174)	47.6 (60/126)^†^	31.8 (90/283)	35.9 (85/237)
LBW (%, n/n)	14.6 (67/458)	17.9 (66/369)	23.0 (40/174)	30.2 (38/126)	9.5 (27/283)	11.4 (27/237)
PTD (%, n/n)	17.8 (81/454)	20.3 (74/365)	24.7 (43/174)	28.6 (36/126)	13.6 (38/279)	15.8 (37/234)

*, *P* = 0.02; ^†^, *P* = 0.005.

## Discussion

In the present study from Ghana, almost two in three women had evidence of present or past placental *P. falciparum* infection. In this highly endemic setting, blood group O was associated with protection against placental malaria among primiparae but not in multiparae, and particularly against late or chronic infections, i.e., infections characterized by the presence of pigment. This finding accords with the clearly protective role of blood group O in severe malaria [5-8] and the moderately beneficial impact of blood group O in many studies on uncomplicated malaria (reviewed by [3,4,9]). Severe childhood malaria and malaria in pregnancy differ largely in terms of pathophysiology, immunity and clinical manifestation [10-12], but primiparae to some extent resemble young children with respect to a lack or an insufficient degree of protective immune mechanisms against *P. falciparum*. In pregnant women, *P. falciparum*-infected erythrocytes sequester in the intervillous space by adhering to ligands on the syncytiotrophoblast, followed by the local accumulation of haemozoin and inflammatory cells. Specific antibodies capable of blocking parasite adhesion prevent this placental malaria only after successive pregnancies exposed to *P. falciparum* infection [11,24,25]. This likely explains why protection against *P. falciparum* infection due to blood group O in the present study was limited to the relatively immune-naïve group of primiparae. At higher parity, the effects of adaptive immunity may override the protection afforded by blood group O.

Notably, however, two studies from The Gambia and Malawi have reported results contrasting the present finding, i.e. an increased risk of active placental malaria in blood group O primiparae and a reduced risk in multiparae [13,14]. The reason for this disagreement is unclear but may involve the classification of placental malaria by histopathological diagnosis in these previous studies and by blood film microscopy and PCR in the present. Remarkably, in both previous studies, past infections (presence of pigment only) tended to be reduced in blood group O primiparae. In the present study, much of the observed associations was based on the lowered presence of haemozoin but microscopically visible parasites also tended to be reduced in group O primiparae (Table 3). Sensitive PCR assays in the present study, considered to detect only viable parasites [26], only shifted this difference between blood groups to a higher prevalence level and, interestingly, the reduced infection rate in blood group O primiparae was observed for both microscopic and submicroscopic infections. This is in line with the lack of effect of the blood groups on parasite density observed in the present study. Part of the conflicting results with and between previous studies may result from small samples sizes [13,15,16] as well as from low prevalence of infection [15-17] in previous studies. Notably, however, in one small study from Gabon, blood group O tended to protect against placental malaria, with a larger effect in primiparae than in multiparae [16]. In almost 1,500 women in Thailand, weekly malaria screening by peripheral blood films did not show any effect of the ABO blood groups on malaria episodes (occurring in 30% of women) [17]. However, peripheral blood film microscopy in pregnant women has a notoriously poor sensitivity [27], only 1% of placentas were malaria positive, and the majority of malaria episodes were due to *Plasmodium vivax*. Therefore, it is questionable whether these findings from Thailand can be compared with the African studies. Blood group O in the present study was also associated with reduced odds of anaemia, particularly in primiparae. Protection from malaria-related anaemia in individuals with blood group O has been observed before [28] but, remarkably, this was not the case in the present study, in which, rather, anaemia was less common among non-infected blood group O women. This finding may reflect the cumulative effect over time of reduced malaria in blood group O primiparae. Longitudinal studies will be needed to address this question.

As a limitation of the present study, protection by blood group O was statistically significant only when considering placental haemozoin or when combining results of all diagnostic means (although microscopic data showed accordant trends). Statistically significant findings were observed in the relatively small group of 300 primiparae of whom 174 had blood group O. The study was sufficiently powered to detect an influence of blood group O on present or past infection but power declined with declining diagnostic sensitivity, i.e. prevalence of infection. Limited sample sizes in subgroups may thus have interfered with the analysis of infection risks in individual genotypes and of potential modification of malaria-related outcomes, e.g., LBW. Also, data on some potentially interfering factors were not available, e.g., bed net use or transfusions. This should be kept in mind when interpreting the data.

The hypotheses on why blood group O protects from malaria, and from severe malaria in particular, include aspects of differential attractiveness to *Anopheles* vectors, shared ABO antigens with *P. falciparum*, impaired merozoite penetration as well as reduced cyto-adherence (reviewed by [3,4,9]). The A and B antigens are erythrocyte surface trisaccharides [22] and receptors for rosetting, i.e. the binding of *P. falciparum*-infected red blood cells (RBCs) to uninfected erythrocytes (an *in vitro* sign of parasites causing severe malaria) [5]. Binding of the parasite ligand, PfEMP1, to the A antigen has been shown [29]. In blood group O, the disaccharide H antigen is produced instead of A and B antigens [22], and *P. falciparum*-infected RBCs form smaller and less firm rosettes than in non-O RBCs [29]. In fact, the protective effect of blood group O against severe malaria has been shown to operate through the mechanism of reduced *P. falciparum* rosetting [5]. However, rosetting is rarely observed in placental *P. falciparum* isolates [30] and the PfEMP1 domain mediating binding to ligands on the syncytiotrophoblast differs from the binding domain involved in rosetting [29,31,32]. Nevertheless, ABO-dependent differences in cyto-adherence may still be relevant in placental malaria (reviewed by [4]): The molecules on the syncytiotrophoblast to which pregnancy-specific *P. falciparum* strains bind, i.e. chondroitin sulphate A (CSA) and hyaluronic acid [33,34], are structurally related to the A antigen which gives rise to the possibility that adhesion of infected RBCs to the syncytiotrophoblast may be influenced by ABO polymorphisms. Also, the placental proteoglycan thrombomodulin mediates part of the binding of infected RBCs [35]. Thrombomodulin and other molecules involved in cyto-adhesion, e.g., von Willebrandt factor, are affected by the ABO phenotype [36]. Recently, enhanced macrophage-mediated phagocytosis of *P. falciparum*-infected O-RBCs has been shown [37]. Considering that many women with placental malaria show a substantial inflammatory infiltrate in the intervillous space, composed mainly of monocytes and macrophages [38], ABO-dependent clearance of infected RBCs could partially explain the finding of the present study of less placental malaria in women with blood group O.

## Conclusion

The present study demonstrates a protective influence of blood group O against *P. falciparum* infection in primiparae living in an area of high malaria endemicity. Considering the consequences of placental malaria in terms of infant mortality, the influence of the ABO polymorphism on malaria in pregnancy may contribute to the high frequencies of the O allele in sub-Saharan Africa.

## Competing interests

The authors declare that they have no competing interests.

## Authors’ contributions

GBA, KT, and FPM designed the study. GBA, TAE and FPM were responsible for patient recruitment, clinical and laboratory examinations. PPG and SM did the PCR analyses, TAE and KT contributed to data interpretation, and FPM did the statistical analyses. GBA and FPM wrote the paper with major contributions of the other authors. All authors read and approved the final manuscript.
